# Skills Capacity Building For Health Care Services and Research Through the Sickle Pan African Research Consortium

**DOI:** 10.3389/fgene.2022.805806

**Published:** 2022-06-15

**Authors:** Obiageli Eunice Nnodu, Alex Osei-Akoto, Victoria Nembaware, Jill Kent, Maxwell Nwegbu, Irene Minja, Gaston Kuzamunu Mazandu, Julie Makani, Ambroise Wonkam

**Affiliations:** ^1^ University of Abuja, Abuja, Nigeria; ^2^ Centre of Excellence for Sickle Cell Disease Research and Training, University of Abuja, Abuja, Nigeria; ^3^ Department of Haematology and Blood Transfusion, College of Health Sciences, University of Abuja, Abuja, Nigeria; ^4^ Department of Child Health, Komfo Anokye Teaching Hospital, Kumasi, Ghana; ^5^ Kwame Nkrumah University of Science and Technology, Kumasi, Ghana; ^6^ Division of Human Genetics, Faculty of Health Sciences, University of Cape Town, Cape Town, South Africa; ^7^ Muhimbili University of Health and Allied Sciences, Dar es Salaam, Tanzania; ^8^ Department of Chemical Pathology, College of Health Sciences, University of Abuja, Abuja, Nigeria; ^9^ Department of Restorative Dentistry, Muhimbili University of Health and Allied Sciences, Dar es Salaam, Tanzania; ^10^ African Institute for Mathematical Sciences, Faculty of Science, Stellenbosch University, Muizenberg, South Africa; ^11^ African Institute for Mathematical Sciences, Cape Town, South Africa; ^12^ Department of Genetic Medicine, John Hopkins University School of Medicine, Baltimore, MD, United States

**Keywords:** capacity building, health care, research, data management, sickle cell disease, Africa

## Abstract

Skills development, the building of human capacity, is key to any sustainable capacity building effort, however, such undertakings require adaptable and tailored strategies. The Sickle Pan-African Research Consortium (SPARCo) is building capacity in sickle cell disease (SCD) management and research in sub-Saharan Africa, including a multi-national SCD patient registry, this is underpinned by skills development activities in data, research, and SCD management.

**Method:** The SPARCo Skills Working Group was set up with the mandate of coordinating skills development activities across the three SPARCo sites in Ghana, Nigeria and Tanzania. To tailor activities to the requirements of the consortium, a needs assessment was conducted at the start of the project which identified skills required for SCD management and research and catalogued existing external and internal training programmes. The needs assessment highlighted differences in skill levels between the sites and different organisational structures which required tailored skills development activities at individual, site and consortium levels.

**Strategy:** Based on the needs and the resources available, different types of training activities were implemented: these included online, blended and face to face activities. In order to create a sustainable skills development programme, existing short, medium, long-term, on-job training activities were used wherever possible. World Sickle Cell Day (19th June) was leveraged for training and health education activities.

**Results:** SPARCo has recorded 1,726 participants in skills development activities across the three sites. Skills have been enhanced in data management, SCD and research to underpin the core deliverables of SPARCo.

**Conclusion and Lessons Learned:** The baseline needs assessments and continual review and adjustment were critical for development of an effective skill development strategy for the consortium. This adaptability was particularly valuable during the COVID-19 pandemic. The sustainability plan leveraged existing programmes and activities and has created a pool of people with required skills for health care and research in SCD. To be effective, skills development programmes need to take into account existing capacity, training opportunities and local conditions. The model was applied to SCD and is adaptable to other skills development in healthcare and research in low and middle- income countries.

## Background

The Sickle Pan African Research Consortium (SPARCO) and the Sickle Africa Data Coordinating Centre (SADaCC) set out to develop a multi-national standardised electronic patient consented database of sickle cell disease (SCD) patients in sub-Saharan Africa (SSA) (Makani et al., 2017; Wonkam and Makani, 2019). The success of this initiative would depend on the concurrent development of skills in SCD healthcare and research within an acceptable ethical, legal and socially acceptable framework to support research and improve healthcare for SCD (Makani et al., 2017; Munung et al., 2019). SPARCO and SADaCC, funded by the United States (United States) National Heart, Lung and Blood Institute (NHLBI) (Grant Numbers U24HL13045881 the Sickle Pan African Research Consortium (04/01/2017–03/31/2021) and U24HL135600 and Sickle Data Coordinating Center, (04/01/2017–03/31/2021)] started in May 2017 with sites in Tanzania (Muhimbili University of Health and Allied Sciences - MUHAS), Nigeria (University of Abuja), and Ghana (Kwame Nkrumah University of Science and Technology) with the aim of registering 13,000 SCD patients by 2021. Coordination was provided by SPARCO Hub, located at MUHAS, and the Sickle Africa Data Coordinating Center (SADaCC) based in the University of Cape Town (South Africa) which is the administrative centre coordinating data standardisation and supporting communications. Coordination between the three sites was achieved through five working groups representing the consortium’s areas of focus: Database and Registry, Health and Standards of Care, Research and Skills supported by a Management Committee (MC) Principal Investigators (PIs) and working group leads. The MC in turn reports to a Steering Committee which includes the Program Officer from the NHLBI, the PI of SPARCO, PI of SADaCC and the Principal Investigators from Ghana, Nigeria and Tanzania (Makani et al., 2017; Makani et al., 2020). The Steering Committee met monthly with responsibility for coordination and management of the project.

### Database and Registry

SPARCO will generate a vast amount of data (demographic, clinical, social, laboratory and genomic data) hence the need for capacity in data collection, database management, data analysis and a standardized terminology for SCD in an ontology (Adekile et al., 2019). In addition to SPARCO, other initiatives such as the Sickle Cell Genomics Network of Africa (SickleGenAfrica) and the Consortium for Newborn Screening (NBS) in Africa for Sickle Cell Disease (CONSA) program of the American Hematology Association (Ghana, Liberia, Kenya, Nigeria Tanzania, Uganda, Zambia) will substantially add to the amount of data available on SCD patients in the near future (Ofori-Acquah, 2020).

### Health and Standards of Care

Patients with SCD require multidisciplinary care, which is not readily available in most settings in SSA. Even when available, care is often limited to the tertiary level of healthcare which may not be accessible as the first point of call by a significant number of SCD patients (Noubouossie et al., 2016). The management of SCD-related complications is highly variable both between and within countries in SSA. SPARCO is creating multi-level Standards of Care for use across SPARCO sites to provide consistent and best practice care for SCD patients in order to strengthen capacity in SCD management.

### Research

There are many unknowns about the prevalence, clinical manifestation and disease progression of SCD in SSA (Williams, 2016). To undertake research to better understand these issues, more skills in epidemiology, genetics and molecular diagnosis, clinical and laboratory phenotyping of SCD, clinical trials, research management, scientific communications, data management and bioinformatics, standardization of specimen collection, processing and shipment, good laboratory practice to minimize variation in preanalytic, analytic and post-analytic processes are required on the continent (Makani et al., 2020).

At the conception of the project, it was recognised that SPARCO sites had some skills gaps in data management, healthcare and research. It was, therefore, proposed to address these skills deficits at undergraduate and specialist levels to involve all cadres of health workers including doctors, nurses, laboratory scientists, community health extension workers (CHEWS), counsellors, health planners and policymakers as well as patient and community groups.

## Methodology

### A. Setting Up of Working Groups

The Skills Working Group (SWG) was formed with the mandate of coordinating skills development activities to support the other specific aims of SPARCO, which are to develop: i) database of SCD patients attending clinics at participating centres, ii) guidelines for locally-appropriate standards of care and iii) plans for research projects. The SWG includes two representatives from each site, the Site Skills Coordinator, a representative from SPARCO Hub and from three from SADaCC. The Chair and a Co-Chair of the SWG are the site PIs for the Nigeria site and the co-PI for the Ghana site, respectively.

The SWG meets monthly, using online video conference platform with face-to-face meetings at the twice-yearly consortium meetings.

### B. SWG Plan Guide

Identification of deliverables and identification of skills priority areas The main deliverables of the SWG were the review of existing skills development programmes, the development of short and medium-term skills training programmes and participation in independently funded training programmes. A work plan was developed to guide activities. Needs assessment was carried out by consortium members. Each site reviewed its own existing skills development programmes, developed training materials/schedules and curricula and these were collated and followed by an assessment of the strengths and weaknesses as well as those common to all. This enabled the SWG to determine the skills required to achieve the aims of the consortium and identify priority training areas to support the work as listed in Table 1.

**TABLE 1:** Selected courses identified in priority areas.

Program	Category of program	Institution	Country/Region
ASH Consultative Hematology Course	Clinical	American Society of Hematology	United States
ASH Visitors Training Program	Clinical and Research	American Society of Hematology	United Kingdom
Haematology Short Course	Clinical and Research	University of West Australia, School of Pathology and Laboratory Medicine	Australia
Laboratory Aspects of Haemoglobinopathy Diagnosis	Clinical	Imperial College, London	United Kingdom
Haematology in Obstetrics Course	Clinical	British Society for Haematology	United Kingdom
Clinical Research Training in Hematology (CRTH)	Research	European Hematology Association	Europe
Graduate Certificate in Cancer and Haematology Nursing	Clinical	University of Sydney	Australia
Blood Science MSc (Distance Learning)	Research	London Metropolitan University	United Kingdom
Master of Science (Msc) electronic (e)-Learning course in Haemoglobinopathies	Research	University College London	United Kingdom
ESH-ENERCA Training Course on Haemoglobin Disorders: Laboratory Diagnosis and Clinical Management	Clinical and Research	European School of Haematology	Europe (Past)
Renzo Galanello Fellowship Programme 2018	Clinical and Research	Thalassaemia International Federation (TIF)	United Kingdom
Module in haemoglobinopathies	Clinical	London Metropolitan University	United Kingdom
Haemoglobinopathies: short counselling course	Clinical	Royal College of Midwives	United Kingdom
Genetic risk assessment and counselling module (Sickle Cell and Thalassaemia Screening)	Clinical	Royal College of Midwives	United Kingdom
T32 Training Program in Hematology	Research	Division of Hematology, John Hopkins University	United States
Hematology Training Program	Research	Boston University Medical Center	United States
Hematology and Oncology Fellowship Program	Clinical and Research	Boston University Medical Center and the Boston VA Health Care System	United States
Hematology Training Program at Herbert Irving Comprehensive Cancer Center	Clinical and Research	Herbert Irving Comprehensive Cancer Center, Columbia University Medical Centre	United States
Haematology and Transfusion Science MSc	Clinical and Research	Manchester Metropolitan University	United Kingdom
Haematology	Clinical and Research	University of Nottingham	United Kingdom
Haematology MSc (by research)	Research	University of Nottingham	United Kingdom
Biomedical Blood Science MSc, Postgraduate Diploma, Postgraduate Certificate Transfusion, Transplantation and Tissue Banking MSc	Clinical and Research	University of Keele	United Kingdom
University of Westminster, London	Clinical and Research	University of Edinburgh	United Kingdom
Biomedical Science (Haematology) MSc	Clinical and Research	University of Wolverhampton	United Kingdom
Biomedical Sciences (Haematology) MSc	Clinical and Research	University of Westminster, London	United Kingdom
Blood Sciences MSc (PGCert PGDip)	Clinical and Research	University of Brighton	United Kingdom
Blood Science (Distance Learning) MSc	Clinical	London Metropolitan University	United Kingdom
Blood Science - MSc	Clinical	London Metropolitan University	United Kingdom
Haematology MSc	Clinical and Research	University of Chester	United Kingdom
Advances in Haematology in Africa	Clinical	Muhimbili National Hospital and Muhimbili University of Health and Allied Sciences	Tanzania
MMed Haematology (10,250,281)	Clinical	University of Pretoria	South Africa
Clinical Haematology Unit CMJAH	Clinical	The University of Witwatersrand, Joburg	South Africa
Advanced Postgraduate Diploma Course in Clinical Research and Data Management	Research	James Lind Institute, Switzerland	Switzerland

### Training Programs

#### Identification of Programs

With the knowledge of training gaps and needs so identified, the SWG decided to seek for programmes/courses where individuals could be trained in these areas. A search on the internet using search terms like haemoglobinopathies, Sickle Cell Disease, Haematology, and others, was done to identify training institutions and programmes that are aligned to the identified needs. Existing partners were also identified who could provide expertise in these areas.

#### Identification of Training Materials

Training material on database management, healthcare, health education and research related to SCD were compiled in both soft and printed versions from all the three sites.

#### Identification of Participants

The skills development activities targeted a health care worker, i.e., doctors, nurses, pharmacists, medical laboratory scientists and community health extension workers, patients and parents.

Where SPARCO had identified gaps in training in priority areas, new training courses and curricula were developed in data, healthcare and research.

### C. Working Group Procedures

Each consortium country had a SWG which meeting regularly and delivered skills development training activities on weekly/monthly basis at site level. Initially these were limited to face to face but with the COVID pandemic and onset of online meeting platforms, workshops, seminars were held which were open to individuals from other consortium sites and countries. Monthly pan consortium meetings took place to report on progress against planned targets. Biannual reports were given.

**FIGURE 1:**
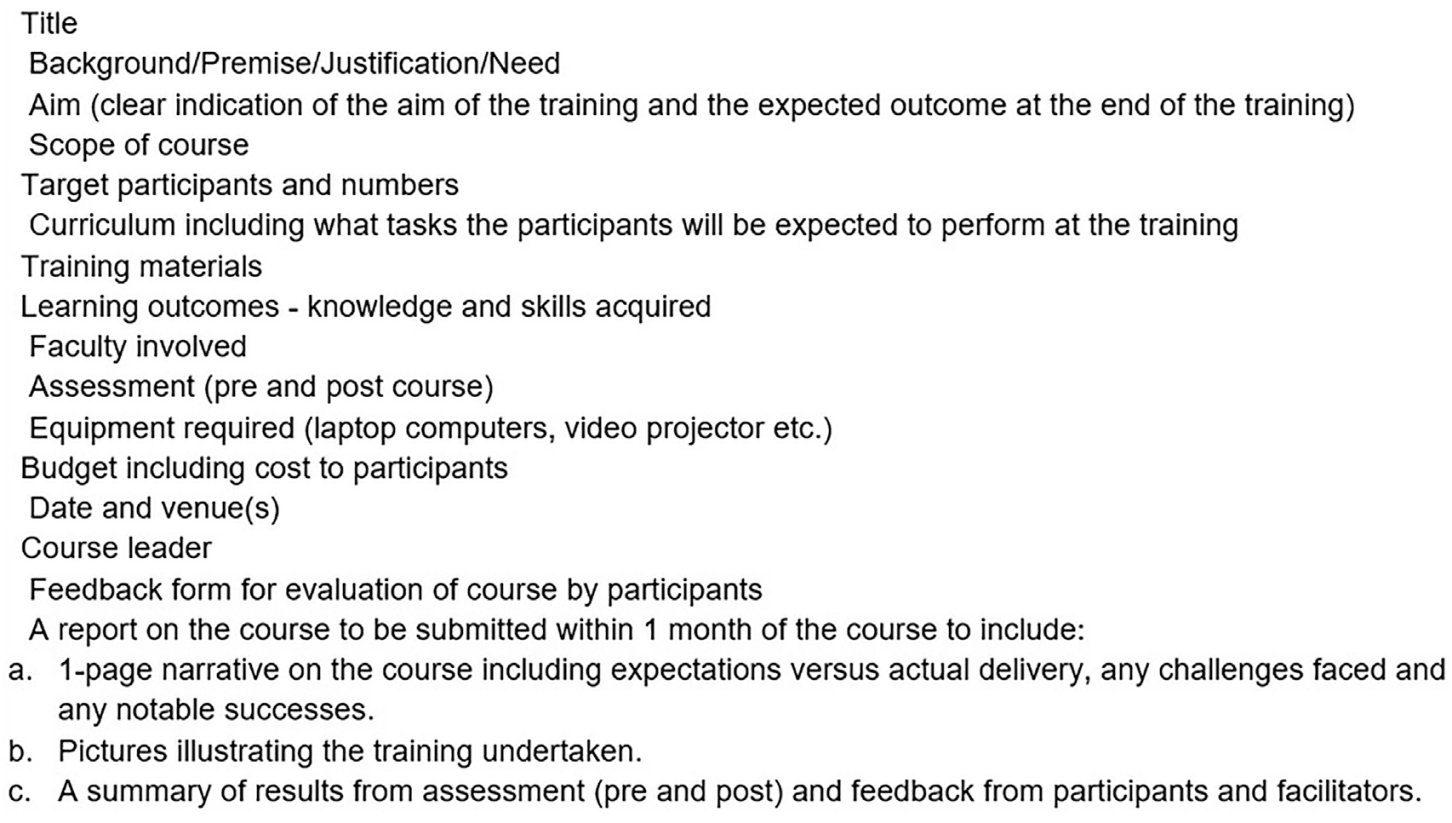
Training SOP.

Workshops were conducted according to the following standard operating procedure which was filled and submitted to SADACC in order to advertise the workshop to a wider audience at the Consortium website (https://sadacc.org/). The Details of the SOP for Training Workshop can be seen in Figure 1. The monthly SWG consortium meetings provided opportunities to share activities from each site with a healthy exchange of ideas and learning from each other.

### D. Training Program Development

Based on the gaps identified, resources and expertise available within the consortium and collaborating partners, the following training programs were developed:

## Data

### Data Collection

**FIGURE 2:**
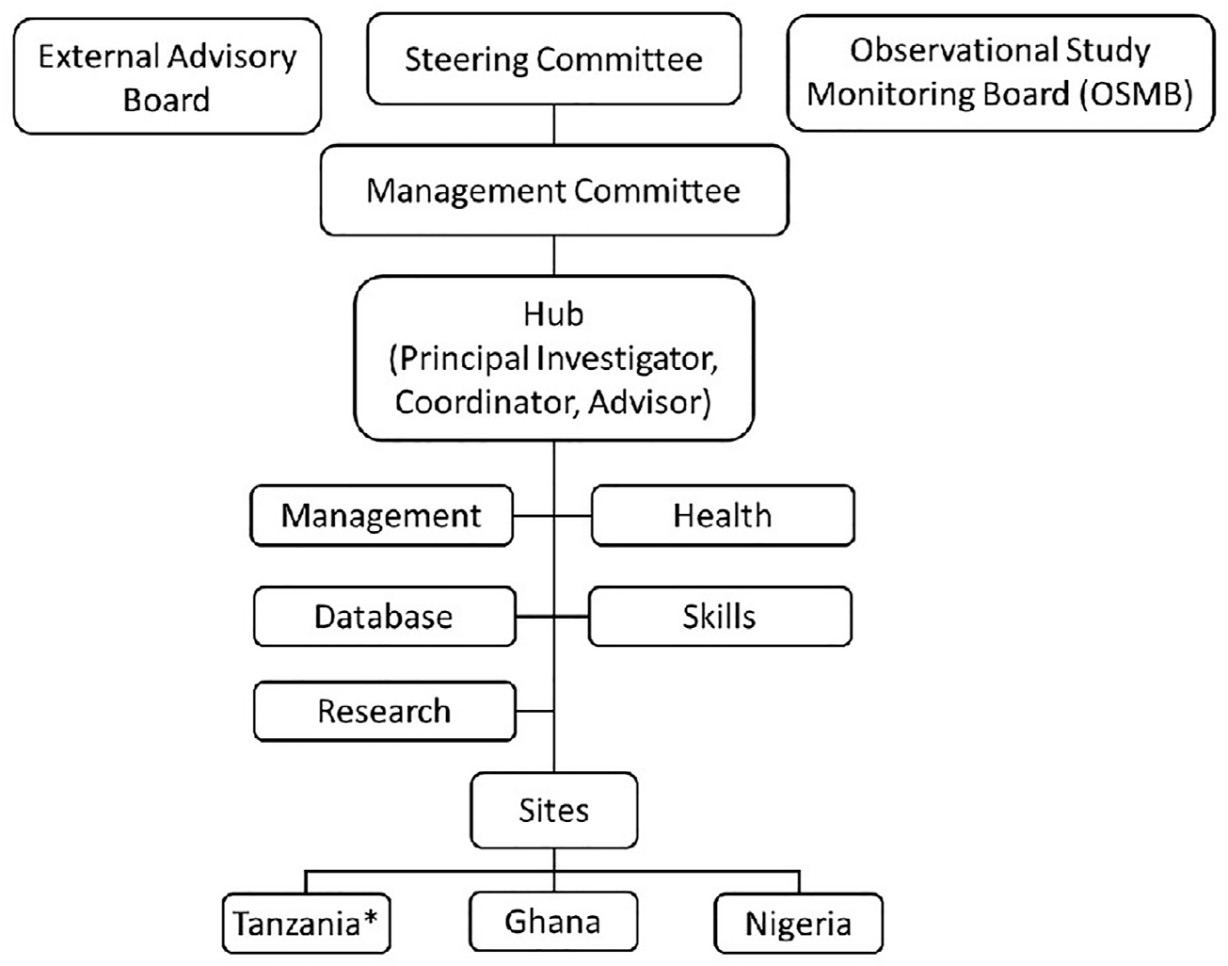
The Organizational Structure of the Sickle Pan African Research Consortium (SPARCO).

Skills in data collection and data management are central to the aim of SPARCO to collect data on SCD patients (Figure 2) and so training in data management was prioritized taking advantage of differing skill levels at different sites and amplifying these by virtual delivery and face to face workshops. SPARCO and SADaCC developed and provided training in data collection instruments and management focusing on REDCap, big data analytics, epidemiology and study design to help address the increasing demands for big data analytics and public health skills for pan-African audiences (Nembaware et al., 2020). The curricula were tightly aligned with the SPARCO goal to implement a SCD database for a large multinational African cohort. The training was designed for the African context, taking into account the constraints of poor access to resources, insufficient computer resources and unreliable internet connections even though the training would ideally be highly computer-intensive with strong and reliable internet connections.

### Study Design

The Consortium sites had been involved in the care of patients with SCD and had their own data collection instruments with different data elements. One of the first things done was to compare these data collection instrument and develop basic data elements for transfer linked to the Sickle Cell Ontology (SCDO) The research assistants were then trained in the use of uniform case report forms (CRFs) for enrolment of patients into the SPARCO registry and for active follow up of patients enrolled in the registry.

### Quality Assurance and Accuracy

Standard operating procedures (SOPs) were developed for use by the Consortium sites. These SOPs covered user name and password management for the electronic registry, user rights and data access groups management, data recording, data receipt, entry and validation, database design, lock, export and archiving, data transfer from sites (depending on the data agreement) patient recruitment and informed consent processes.

### Data Collection and Management Training

A data collection and management workshop curriculum were developed and implemented in a competency-based manner (manuscript in development). The curriculum was designed to become incrementally more advanced with the aim of enabling trainees to build data collection instruments, store clinical records or case report forms containing SCD patients’ medical and environmental information using the research electronic data capture (REDCap) platform and ensure high quality database and export and import datasets.

### Quality Assurance Processes and Data Quality Checks

Data quality and assurance are a major component of the data management pipeline and therefore training was provided in data quality assurance processes and data quality checks to assess accuracy, completeness, consistency, integrity, validity, timeliness and ethical integrity. Data management and quality assurance Standard Operating Procedures (SOPs) were created and sites were trained on how to implement these SOPs to improve data quality across the three sites.

### Training in Big Data Analytics SCD and Epidemiology Study Design

The main goal of the Big Data analytics course was to create a cohort of individuals, Big Data Analytics Fellows, with the skills to analyse big data sets such as those being generated at SPARCO sites: specifically, data cleaning, analysis, and study design, from a Big Data perspective, using the SPARCO database. The training covered fundamental concepts of probability, statistics and programming in R, which includes sampling and estimation theory, logic of inferential statistics, testing hypothesis, statistical power calculation and sample size estimation. To build generic research skills and provide fellows with the skills to interrogate the literature and identify gaps in the knowledge base, training on systematic reviews was provided, the systematic reviews resulting from this exercise will be the foundation of crossconsortia studies led by these young investigators.

## Health

### SCD Management

Advances in Haematology in Africa was a 4-day workshop held in Tanzania in August 2018 designed to build skills and knowledge in haematology, specifically focussing on the African context. The course included a day devoted to SCD and was attended by members of SPARCO Hub and site teams. The trainers were experts in haematology from Africa and outside Africa with funding, including bursaries to assist attendance, provided by the Fondazione Internazionale Menarini, Italy. Additionally, the Standards of Care (SoC) Working Group, another working group of SPARCO developed Standards of Care Guidelines for the clinical management of SCD patients. These guidelines are also being used for training of health care workers in the multidisciplinary management of SCD at all levels of health care including home care management.

### Health Education

SPARCO recognizes health education as one of the key components for the execution, implementation, and success of its agenda in the three key areas: establishing a patient registry, generating locally appropriate standards of care and research. To help achieve these, sites have to provide health education in SCD engaging with key stakeholders and communities using all available existing structures and platforms.

## Research

### Target

The target of SPARCO’s research training were not only physicians in laboratory medicine internal medicine, community medicine but also basic medical scientists, pharmacists, nurses, medical laboratory scientists, laboratory technicians, medical records officers and community health extension officers.

### Core Intercalating SCD Training in Existing Programmes

During the term of the project, SPARCO sought to build skills by building on utilising existing training or developing training programmes where gaps in provision were identified. As proposed, SPARCO made use of local strengths and resources available across the different regions by keying into existing programmes such as the Fellowship of the West African College of Physicians (WACP) and College of Pathology for East Central and Southern Africa (COPECSA) and the African Society of Laboratory Medicine (ASLM) in partnership with the American Society of Hematology, (2021), Royal College of Pathologists and Physicians in London and the European Hematology Association (EHA) as well as collaboration with the Medical Education Partnership Initiative (MEPI), and Human, Heredity and Health in Africa (H3Africa). It was envisioned that the incorporation of sub-specialization in SCD in disciplines such as haematology and paediatrics the WACP and COPECSA will lead to the creation of SCD units/centres in hospitals to be overseen by specialists trained by these programs thereby enhancing skilled manpower within these settings. A second platform for long term skill development is postgraduate academic programs in the tertiary institutions within the network. The consortium is working to infuse advanced training in SCD into these existing programs such as Masters level degrees in haematology and clinical research.

Consequently, a Masters (MSc) in Clinical Research 2-year programme, was developed at the University of Abuja, Nigeria site. It is targeted at physicians in laboratory medicine, internal medicine, general practice and other specialties to enable them undertake research geared towards solving health care problems. Core courses include biostatistics, epidemiology, fundamentals of clinical trials, scientific communication, with options modules to allow for specialisation including haemoglobinopathies, diagnostic pathology and transfusion medicine. It is planned to offer the course online to make it available across the continent, including other SPARCO sites.

### Training Modalities

The training modalities employed have been Train -the -Trainer through data management training workshops by SADACC at the University of Cape Town, face to face training through lectures, seminars, workshops. These were supplemented by use of e-learning platforms for genetic counseling, ethics in health research and webinars as a result of the corona virus disease pandemic.

### Long Term Skills Development Plan

The SPARCO Fellowship programme was initiated in the fourth year. Early-career scientists were invited by the sites to apply for funds to undertake research in SCD tailored towards SPARCO priority areas with mentored guidance from site members. They were guided in this process by the use of the project concept documents earlier developed by the consortium. The Fellowship project is a mentored research training in research, scientific communication and publication.

Aside physicians, the need to train other cadres of healthcare workers such as nurses, laboratory scientists, community health extension workers (CHEWs), etc. is also part of the long-term skill development plan. The training of the latter will ensure effective NBS biosampling, SCD diagnosis and basic care for SCD patients especially at the primary healthcare level. It is generally accepted that at this level of care, patients are initiated to the concept of active participation in their care. Whereas self-management driven by the concept of behavioural adaptation (BA) has been pushed for adolescents and young adults with SCD, as drivers for overall efficacy in management, there is a need to inculcate these BA skills appropriately for optimal care delivery.

Another important factor for training targeted staff at this level of healthcare delivery, is to create effective coordination and liaison between the patient and the SCD specialists, commonly restricted to tertiary level practice, for management beyond these levels of primary care.

### Participation in Independently Funded Training Programmes

A key component of the SPARCO approach has been leveraging on existing training programmes in African Universities and professional training programmes within the African continent as a sustainable method of providing training past the end of the SPARCO funding. One of the primary activities of the SWG is to facilitate the participation of SPARCO staff in independently funded training programs. During the last year of the project, some SPARCO members participated in the ARISE project training workshops as faculty.

## Results and Discussion

### Overall Achievements

#### Patient Registry

By the end of March 2021 (end of the research), the consortium had registered in the database at the three sites a total of 13,170 SCD patients (Tanzania 3,594; 90% of target of 4000., Nigeria 6,453, 107.6% of target of 6000 and Ghana 3,146, 104.9% of target of 3000). This clearly shows that the target for the aim of the registration was fully achieved and exceeded within the time frame for the project.

#### Data Management

In the first 3 years of SPARCO 277 people across all sites in the consortium participated, including data clerks, data coordinators, doctors, laboratory scientists, researchers and postgraduate students received data management and analysis training.

#### Training in Big Data Analytics SCD and Epidemiology Study Design

With 20 registered candidates and 10 invited trainers, representing a ratio of approximately 2:1 trainee-trainer, training was provided in person, virtually and in a blended format through workshops, site visits, mentorship, monthly meetings and during annual face to face consortium meetings.

#### Identification of Priority Training Areas (Needs Assessment) and Review of Existing Training Programs

During the period 2017–2018, the SWG reviewed existing training programmes to identify relevant programmes and significant gaps in training opportunities in SPARCO priority areas (Table 1). SWG identified and reviewed 41 existing training programmes around the world and 31 were found to be relevant and aligned to the consortium priority areas: data management, management of SCD, health education and research on SCD (Table 2). Gaps were noted in existing programmes in areas related to laboratory diagnosis, NBS, genetic counselling and research. Twenty-eight (i.e, 90.3%) of training programmes/courses identified were located outside of Africa. Areas where gaps were identified, were noted in the development of short, medium- and long-term courses related to SPARCO priority areas. During the project, the SWG undertook continual needs assessment taking into consideration the developments in the project environment and implemented skills development activities, created training materials and identified courses based on these new needs. For instance, in 2018, when the SCD clinical phenotypes from 3,622 patients in the SPARCO Nigeria registry were analysed, it was noticed that hydroxyurea utilisation was 9.4% while blood transfusion rate was 67.5%, showing a gap in the use of hydroxyurea. These results prompted the development of webinars that focused on hydroxyurea (Isa et al., 2020). Similarly, another Webinar on gene editing was carried out as interest in gene edited arose as a result of clinical trials on gene editing.

**TABLE 2:** Selected courses identified in priority area.

Category	Programme and Institution	Content	Country	Target Population
Clinical	1. ASH Consultative Hematology Course American Society of Hematology	This is a practical programme involving commonly encountered clinical problems in which participants engage in case-based presentations and have the opportunity to interact with haematology experts	United States	Clinicians-mainly Physicians
	2. Laboratory Aspects Of Haemoglobinopathy Diagnosis Imperial College, London	Participants learn the practical aspects of haemoglobinopathy diagnosis including microscopy, blood count interpretation, cellulose acetate and agarose gel electrophoresis and high-performance liquid chromatography.	United Kingdom	Physicians, Laboratory Scientists
	3. Haematology in Obstetrics Course British Society for Haematology.	An Interactive and informative programme providing up-to-date insights from experts in the field. The course is suitable for any health care professional interested in this field.	United Kingdom	Consultants or registrars in obstetrics, haematology or obstetric anaesthesia, senior nurses, midwives, scientists, and pharmacists
	4. Module in haemoglobinopathies London Metropolitan University	Course provides an understanding and knowledge of the theory and practice of haemoglobinopathy screening, diagnosis and ethical issues arising, aetiology, Epidemiology, Genetics and Pathophysiology of Haemoglobinopathies (focusing on Thalassaemia (Thal) and Sickle Cell (SC)) Counselling, management, and treatment considerations	United Kingdom	Physicians, scientists, Genetic counsellors etc
	5. Haemoglobinopathies: short counselling course Royal College of Midwives	Short counselling courses for health professionals who care for families affected by sickle cell disease or thalassemia. Particularly valuable for staff caring for people affected by sickle cell disease or thalassemia.	United Kingdom	Nurses, midwives, scientists, Genetic counsellors
	6. Genetic risk assessment and counselling module (Sickle Cell and Thalassaemia Screening) Royal College of Midwives	Course is to: help develop an understanding of the antenatal and newborn sickle cell and thalassaemia screening programme, gain a basic knowledge of sickle cell and thalassaemia and how these conditions are inherited, learn how to interpret screening results and Genetic risk assessment and counselling module	United Kingdom	Nurses, midwives, Health Educators etc.
	7. Blood Science (Distance Learning) MSc London Metropolitan University	Programme focuses on the diagnostic techniques, quality assurance/quality control (QA/QC) and regulatory issues within this field. It offers the advantage of the opportunities for knowledge and career development	United Kingdom	Physicians, Laboratory Scientists, Staff of regulatory bodies in lab sciences
	8. Blood Science – MSc London Metropolitan University	Helps students to develop extensive knowledge in the emerging area of blood science	United Kingdom	Scientists
	9. Advances in Haematology in Africa Muhimbili National Hospital and Muhimbili University of Health and Allied Sciences	Training in clinical haematology especially areas of haemoglobinopathies, sickle cell disease	Tanzania	Physicians
	10. MMed Haematology (10250281) University of Pretoria	4-years Training in clinical haematology especially areas of sickle cell disease, haemophilia.	South Africa	Physicians
	11. Clinical Haematology Unit CMJAH The University of Witwatersrand, Joburg	Clinical Haematology provides a consultative service to patients at the CMJAH and Adult Haemophilia Clinics. Registrars rotate through Clinical Haematology. There is also a formal two-year fellowship programme	South Africa	Physicians
Research	1. Clinical Research Training in Hematology (CRTH) European Hematology Association	Provides early career researchers with a 9-month long unique training and mentoring experience focused on clinical research in Europe, with a global scope. It offers improved skills and knowledge in clinical science.	Europe	Scientists, Early career Clinical Researchers, Haematologists
	2. Blood Science MSc (Distance Learning) London Metropolitan University	The course offers extensive knowledge in the emerging area of blood science. Helps to develop high-level reasoning skills and contribute to lifelong learning and continuous professional development (CPD)	United Kingdom	Laboratory scientists
	3. Master of Science (Msc) electronic (e)-Learning course in Haemoglobinopathies University College London	Objective is to teach health care professionals all aspects of SCD and Thalassemia with emphasis on holistic patient care	United Kingdom	Physicians, Scientists
	4. T32 Training Program in Hematology Division of Hematology, John Hopkins University	The Training Program in Hematology provides interdisciplinary laboratory training for postdoctoral fellows preparing for full-time careers in hematology research. The curriculum emphasizes individual research training, incorporates specified courses and seminars, and provides trainees with professional development opportunities.	United States	Physicians, Scientists
	5. Hematology Training Program Boston University Medical Center	“Research Training Program in Blood Diseases and Resources,” T32HL07501. This is an interdepartmental training programme designed to train predoctoral PhD degree candidates and postdoctoral MDs, PhDs, and MD, PhDs in hematology related research	United States	Physicians, Scientists, Haematologists
	6. Advanced Postgraduate Diploma Course in Clinical Research and Data Management James Lind Institute, Switzerland	This programme is designed to provide overview of clinical trials, clinical research management, clinical trial monitoring and data management	Switzerland	Junior Clinical researchers, Research Assistants, Data managers
Clinical and Research	1. ASH Visitors Training Program American Society of Hematology	Designed to help build hematology capacity in low- and middle-income countries by providing funding for hematologists and hematology-related health care professionals in these regions to receive up to 12 weeks of training on a specific topic or technique. Training is designed to address a specific hematology need and is carried out in the clinic or laboratory of an ASH member, under his/her supervision and mentorship	United States	Haematologists and haematology-related healthcare professionals
	2. Haematology Short Course University of West Australia, School of Pathology and Laboratory Medicine,	Short course for participants with advanced qualifications and it is designed to upskill or reskill in the field	Australia	Haematologists,
	3. ESH-ENERCA Training Course on Haemoglobin Disorders: Laboratory Diagnosis and Clinical Management European School of Haematology	Course is to promote and facilitate access to state-of-the-art and cutting-edge knowledge in haematology and related disciplines. Course exposes participants to the current state-of-the-art science and insight into new developments in the fields of basic, clinical, and therapeutic research in Haematology	Europe	Physicians, Scientists, Pharmacists,
	4. Renzo Galanello Fellowship Programme 2018 Thalassaemia International Federation (TIF)	Training in haemoglobinopathies and to become a trainer of others.	United Kingdom	Paediatricians or Internal Medicine physicians already working with patients with Thalassaemia or Sickle Cell Disease
	5. Hematology and Oncology Fellowship Program Boston University Medical Center and the Boston VA Health Care System	An extensive programme of patient care, education, and research in various clinical and basic science research projects.	United States	Clinicians,
	6. Hematology Training Program at Herbert Irving Comprehensive Cancer Center Herbert Irving Comprehensive Cancer Center, Columbia University Medical Centre	Offers training to become genetic counselor with compassion, a sense of self, and the skills to be a leader in genetic and genomic health care.Curriculum combines basic science and clinical medicine with humanism and professionalism Clinical research in many fields	United States	Physicians, Nurses, Geneticists, others.
	7. Haematology and Transfusion Science MSc Manchester Metropolitan University	Course enables development of an advanced theoretical understanding and the practical techniques needed to apply in research or clinical context.	United Kingdom	Clinicians, Scientists
	8. Haematology University of Nottingham	Course trains individuals in peripheral blood stem cells for matched and unrelated donor transplantation	United Kingdom	Clinicians, Scientists
	9. Biomedical Blood Science MSc, Postgraduate Diploma, Postgraduate Certificate University of Keele	Course offers postgraduate training in Biomedical Blood Science in combination with generic higher level scientific training in areas such as writing grant proposals and business plans. The programme builds on existing, undergraduate knowledge in basic science and applying it to clinical, diagnostic and research applications relevant to Clinical Biochemistry, Medical Immunology, Haematology and Transfusion Science	United Kingdom	Clinicians, Scientists
	10. Transfusion, Transplantation and Tissue Banking MSc University of Edinburgh	The course is expected to achieve background academic knowledge and an understanding and the application of this in many fields such as: donation of blood, organs, and tissues; components, reagents, and products - principles and processes; clinical transfusion practice; clinical laboratory practice (as it relates to transfusion, transplantation and tissue banking); transfusion microbiology	United Kingdom	Clinicians, Scientists
	11. Biomedical Sciences (Haematology) MSc University of Wolverhampton	Course focuses on recent advances in genetics and immunology, which equips individuals with skills necessary to complement clinical laboratory responsibilities	United Kingdom	Scientists
	12. Biomedical Sciences (Haematology) MSc University of Westminster, London	This course focuses on the physiology and pathology of blood and its use as a diagnostic and therapeutic tool. Involves a variety of areas of molecular and cellular bioscience with an emphasis on new technologies and developments in Haematology and related disciplines.	United Kingdom	Clinicians, Scientists, Pharmacists
	13. Blood Sciences MSc (PGCert PGDip) University of Brighton	A 2-year part-time course leading to MSc in blood sciences	United Kingdom	Physicians, Scientists
	14. Haematology MSc University of Chester	The course is designed to enable you to develop an up-to-date, advanced understanding of the disorders of blood in Research and Clinical Medicine cores	United Kingdom	Physicians, Scientists

#### Training Evaluation and Outcomes

Course evaluation was mapped to specific learning outcomes, including processing epidemiological data from multi-site observational studies (from cleaning or performing data quality checks to analysing large diverse data sets), drafting a proposal for ethics clearance for a multi-site retrospective study or systematic reviews on designing a multi-site epidemiological study. To assess how well trainees assimilated and understood the online training content, they were tested on their understanding of Linux commands, databasing, statistics and probability, Python and R programming languages, emphasizing on Python SciPy and R tidyverse libraries: the average mark was above 80%. Trainees presented their systematic review at the fourth SCD Ontology (SCDO) workshop in November 2019 (Nembaware et al., 2020). Assessment of quality of training was conducted in a form of a survey using course evaluation forms to capture feedback from trainees. Feedback showed that most of the fellows recommended ongoing training, and highlighted the difficulties with internet connections during the online training. One of the best outcomes was seeing trainees (one at each site) becoming trainers in data management and analysis at their respective sites during the 2019 site visits and working together in the first publication by a big data fellow (Chianumba et al., 2022).

#### Health and SCD Management

Up to 34 Courses were attended or offered by the SPARCO consortium related to the management of SCD involving 1,318 participants in the first 3 years of SPARCO (Table 3). The courses included, clinical management of SCD, genetic counselling, NBS for SCD, hydroxyurea prescription and use in SCD, infections in SCD, drug addiction and psychosocial issues in SCD, health education for health care providers (HCP), genomic medicine and SCD analgesics and adherence to medications. Courses for diagnosis of SCD included: use of laboratory diagnostic platform for SCD including High-Performance Liquid Chromatography (HPLC), Isoelectric Focusing (IEF) and Hemoglobin Electrophoresis (HbE), and Transcranial Doppler (TCD) ultrasonography. Attendance at these courses improved the ability of qualified HCP to offer quality care to patients with SCD in the three sites. Additionally, the Standards of Care Working group (SoC), Standards of Care Guidelines for the clinical management of SCD patients across the various levels of Health care institutions augmented the knowledge and skills of the HCPs in the management of patients at the sites.

**TABLE 3:** Numbers of participants in priority training areas by SPARCO sites.

Skill area	Broad Competence Outcomes	Ghana	Nigeria	Tanzania	Total
Data management	Data Quality Assurance	80	73	124	277
	REDCap & integration with other data tools				
	Big data analysis				
Health - Management of SCD	Multi-level care for SCD	537	569	212	1318
	Primary level				
	Secondary level				
	Tertiary level oIndices and indicators for SCD referrals				
	SCD diagnosis (POCT, HPLC, IEF)				
Research skills	Grant proposal	36	58	6	100
	Scientific writing				
SPARCO Fellowships	Research proposal development on SPARCO priority areas under mentorship	9	8	12	29

#### Health Education

Sites have engaged with key stakeholders and communities to provide health education designed to build knowledge of SCD, the target audiences being patients and their families, healthcare workers, academic and research communities as well as the general population. The 19th June, World Sickle Cell Day, which has been used to commemorate SCD and increase awareness about the disease, is a key part of this approach with events in health facilities and community as well as interviews in the media. SPARCO, recognizing the key role played by community organisations in mobilizing patients to participate in research and engage with healthcare organisations to receive preventative treatment, has identified a skills gap in these organizations and has worked with patients and patient communities to facilitate participation in site activities and provide training and support in project management and developing grant applications. This has been done utilizing both in-person and online approaches.

#### Health Education on Nutrition in Pregnancy and SCD

With increasing survival rates in SCD patients, supporting pregnant mothers with SCD is becoming more important. A short course on Health Education on Nutrition in Pregnancy and SCD was developed and offered to nurses and midwives working at SCD clinics, antenatal and reproduction and child health clinics in Dar-es-Salaam, Tanzania.

#### Research

Research skills development was mainly through participation in independently funded training courses and SPARCO fellowships (12 Tanzania, nine Ghana, eight Nigeria) SPARCO Fellowships have been awarded to date. The Novartis Next Generation Scientist provided research training opportunities to five (5) SPARCO members at all three sites.

There have been 50 publications from the various research works that have been carried out across the three sites (List of publications available upon request). There are other manuscripts in preparation for publication.

#### Lessons Learned

The SPARCO sites and SADACC are embedded in academic institutions with SPARCO personnel holding academic positions and being integrated in academic departments. SPARCO personnel have increased their competencies and skills in health, research and data management and will make a positive impact on the quality of teaching, supervision and mentoring of students at undergraduate and postgraduate levels at the three SPARCO academic institutions.

SPARCO was successful in skills development in SPARCO priority areas of data management, management of SCD and research skills. In data management, a different approach to training was required as it was not always practical or feasible to conduct residential or in-person training across the consortium., Online training methods using prerecorded videos stored in Google for download and self-study, Vula (a learning management system which allowed people to leave questions online when they had internet) were suitable for participants even with limited quality internet which was suitable for participants even with limited quality internet. Within the healthcare sector, three key lessons were learned when delivering skill development activities in SCD management. First, health care workers rotated frequently which meant that there was a need to offer training on a regular basis to new clinical staff. Second, it was not practical to conduct training away from health facilities as the staff were required to provide health services. Third, although health education material can be developed and shared at the consortium level, it needed to be adapted to local situations such as the use of malaria prophylaxis which was not uniformly practiced in some countries. In research, training was best integrated into research projects, allowing skills to be acquired and applied in the context of a project rather than in theory. As well as developing skills in priority areas through participating in SPARCO, the SPARCO team developed skills in non-technical areas such as project management and curricula development, however, specific development of competencies in curriculum development and accreditation and project management would have helped delivery of SPARCO skills development activities.

## Conclusion and Future Plans

Going forward, SPARCO plans to strengthen skills in SCD by engaging with existing training programs in institutions at national, regional and global levels. With the availability of digital education tools and lessons learned in online activities in the wake of the COVID-19 pandemic, SPARCO skills development activities have the potential to draw upon the large pool of expertise from participating consortium and collaborating institutions and other independently funded training programs. This strategy of working with existing training programs to develop and utilise resources that will greatly contribute to raising the skilled workforce required for the management of SCD in all levels of health in SSA countries and to support, basic, clinical and translational research in SCD and is designed to enhance the sustainability of skills development in SCD beyond the end of the SPARCO.

Skills development will focus on the priority areas of data management, management of SCD and research methodology and be aligned to the specific aims of governance, operations, management, SCD Registry and database, Integration of SCD Standards of care and clinical skills, cohort and implementation research skills and engagement with partners. Skills development activities will be delivered to researchers, health care workers, data and registry personnel, faculty in collaborative institutions and hospitals, patients/community, as well as key stakeholders and decision-makers to enhance sustainability. We will respond to emerging skill requirements by identifying or developing training courses. We will collate training courses available at all SPARCO sites, so as to avoid duplication of effort. More effort will be made to have cross consortia skills development activities in order to maximally utilise expertise at local sites for the benefit of all.

## Data Availability

The original contributions presented in the study are included in the article/Supplementary Material, further inquiries can be directed to the corresponding author.
